# Depression and health literacy among adolescents and adults in Germany: findings from two representative samples

**DOI:** 10.3389/fpsyg.2024.1494333

**Published:** 2024-12-11

**Authors:** Lars König, Rebekka Schröder, Tim Hamer, Ralf Suhr

**Affiliations:** ^1^Stiftung Gesundheitswissen, Berlin, Germany; ^2^Institut für Medizinische Soziologie und Rehabilitationswissenschaft, Charité – Universitätsmedizin Berlin, Berlin, Germany

**Keywords:** depression, depressive disorder, health literacy, health, representative, Germany, adolescents, adults

## Abstract

**Introduction:**

Depressive disorders constitute a significant public health challenge. Health literacy might be an important factor to consider in prevention strategies for depressive disorders, which is why this study aimed at exploring the association between depressive symptom levels and severity and health literacy, along with additional sociodemographic factors.

**Materials and methods:**

Data were collected from two large samples of adults (*N* = 3,011) and adolescents (*N* = 1,021) representative of the German-speaking population in Germany. Levels of health literacy (HLS-EU-Q16 questionnaire), depressive symptom severity, rates of depression levels (PHQ-9 questionnaire) and sociodemographic information (age, gender, social status, level of education) were obtained. The associations between sociodemographic factors, health literacy and depression were analyzed using *t*-tests, analyses of variance and χ^2^-tests.

**Results:**

Overall, rates of depression were high in both samples (16.5% in adults and 18.4% in adolescents) when measured with the sum score ≥ 10 cut-off criterion and substantially lower when assessed with the diagnostic algorithm criterion (7.2% in adults and 9.8% in adolescents). Rates of depression and severity of depressive symptoms were higher in female than male individuals in both samples. Depressive symptom severity and depression rates increased with increasing age in adolescents and decreased with increasing age in adults. Higher levels of education and lower social status were associated with higher depressive symptom severity and rates in adults, with a more heterogeneous picture in adolescents. In both samples, depressive symptom severity and rates were higher in individuals with poorer health literacy.

**Discussion:**

The results point to a potential role for health literacy in preventing depressive disorders. More research is needed with longitudinal and experimental study designs into the question whether public health interventions targeting health literacy improvements could play a critical role in reducing the burden of depression across different age cohorts.

## 1 Introduction

Depressive disorders are prevalent psychiatric conditions that affect millions of people world-wide and are associated with substantial burden both at the individual and the societal levels (Chan et al., 2023; GBD 2019 Mental Disorders Collaborators, 2022; Chesney et al., 2014). All depressive disorders are characterized by depressive (e.g., sad, irritable) mood or loss of pleasure accompanied by other symptoms that significantly affect the individual’s ability to function (World Health Organization, 2019).

In Germany, the 12-months prevalence of unipolar depression is 7.7% (Jacobi et al., 2014), which is comparable to the 12-months prevalence retrieved from meta-analyses of global data (Lim et al., 2018). Age of onset for depressive disorders is typically in the mid-twenties, with the peak risk age ranging from mid-to-late adolescence to early forties and a modest decrease in prevalences with older age (Otte et al., 2016; Bromet et al., 2011). Significantly higher prevalences are found in women (10.6%) than in men (4.8%; Jacobi et al., 2014) and incidence rates have increased by almost 50% on a global level from 1990 to recent years (Liu et al., 2020; Moreno-Agostino et al., 2021). This is particularly alarming given that depressive disorders are associated with severe consequences for the affected individuals including adverse health outcomes, physical and mental comorbidities and overall reduced quality of life (Alonso et al., 2004; Kang et al., 2015; Jacobi et al., 2014; Whooley and Wong, 2013). Of note, mortality in patients with depressive disorders is substantially higher than in the general population and the risk of suicide is increased by a factor of 20 in people with major depressive disorder compared to the general population (Chesney et al., 2014). As a result, depressive disorders are considered a major cause of overall burden of disease, e.g., in terms of disability-adjusted life years, both globally and in Germany (Vos et al., 2016; Chan et al., 2023; Plass et al., 2014; Porst et al., 2022; GBD 2019 Mental Disorders Collaborators, 2022). Depressive disorders are even projected to be the leading cause of disability worldwide by 2030 (World Health Organization, 2008). In addition to the impact on the affected individual’s quality of life, depressive disorders are also associated with substantial direct and indirect costs (König et al., 2019).

Etiology of depression is described in terms of a vulnerability-stress model, in which the emergence of the disorder is explained by the interactive interplay of trait-like vulnerabilities (i.e., genetic or biological factors that make a person susceptible to psychopathology) and stress factors such as undesired significant life events or the accumulation of minor life events and socioeconomic factors (Ingram and Luxton, 2005). The heritability of major depression is estimated at about 37% (Sullivan et al., 2000), but results from genome-wide association studies suggest that major depression is highly polygenic and only few potential risk genes with small effect sizes have been discovered so far (Flint and Kendler, 2014; Kendall et al., 2021). A large number of risk factors for depressive disorders have been investigated with mixed results concerning their validity. However, convincing evidence has been found in a recent meta-analysis for widowhood, physical abuse during childhood, obesity, having 4–5 metabolic risk factors, sexual dysfunction and job strain (Köhler et al., 2018).

Treatment of depressive disorders is usually based on a pharmacological or psychotherapeutic approach or a combination of both (Cuijpers et al., 2020; Bundesärztekammer et al., 2022). There is no conclusive evidence for the superiority of one treatment option to another (Bundesärztekammer et al., 2022), but a recent meta-analysis found highest response for combined pharmacological treatment and psychotherapy (Cuijpers et al., 2020). While most evidence is available for cognitive-behavioral therapy, different approaches to psychotherapy were found to yield similar results in terms of efficacy and acceptance (Cuijpers et al., 2021; Barth et al., 2016).

Importantly, the overall burden of the disease can be mitigated not only by successfully treating patients with depression, but also by preventing the onset of depressive disorders (Andrews et al., 2004). Effectively preventing depressive disorders is particularly important considering the global rise in depressive disorders making it a major public health challenge in the upcoming decades (Liu et al., 2020; Moreno-Agostino et al., 2021). Therefore, a number of prevention programs have been developed and employed, with mostly small but positive results, even in cost-effective settings (Bellón et al., 2019; Bellón et al., 2015; Conejo-Cerón et al., 2017; Deady et al., 2017; van Zoonen et al., 2014). For example, eHealth interventions based on cognitive-behavioral approaches have been found to reduce depressive symptoms (Deady et al., 2017).

Health literacy, the cognitive and social skills which determine the motivation and ability of individuals to gain access to, understand and use information in ways which promote and maintain good health (Nutbeam, 1998), has been identified as a major determinant of various health outcomes, including mental health (Berkman et al., 2011). For example, inadequate health literacy has been consistently associated with higher probability of being depressed (Mo et al., 2023; Gazmararian et al., 2000; Lincoln et al., 2006; Guo et al., 2023; Smith and Moore, 2012). Gazmararian et al. (2000) showed in a large sample of elderly adults that those with inadequate functional health literacy had 2.7 times the odds of being depressed according to a structured interview than those with adequate functional health literacy skills. Similarly, Mo et al. (2023) showed that individuals with depression and inadequate health literacy reported higher depression scores compared to those with depression but adequate health literacy. Mechanistically, health literacy might serve as protective factor against the development of depressive disorders by enhancing access and utilization of health care, improving patient-provider relationship and promoting self-care (Paasche-Orlow and Wolf, 2007). In a recent investigation, a targeted health literacy intervention in a sample of participants with low health literacy not only improved various health outcomes but also reduced depressive symptoms (Uemura et al., 2021). However, more high-quality research in this field is necessary. Specifically, risk groups that could particularly profit from health literacy interventions need to be identified.

The aim of the current study was to explore the relationship of depressive symptoms and health literacy in two samples of adolescents and adults in Germany. Through this exploration, the present paper seeks to contribute to a more nuanced understanding of how health literacy relates to depressive symptoms and to inform the development of prevention programs aimed at individuals at particularly high risk for poor health outcomes.

## 2 Materials and methods

### 2.1 Ethical considerations

The study was conducted in accordance with ethical principles for medical research involving human subjects as set out in the Declaration of Helsinki as well as local legislation. The ethics committee of the Berlin Medical Association had no ethical or professional objections to the study protocol (reference Eth-64/23). All participants provided informed consent to take part in the study. In addition, informed consent was obtained from parents or legal guardians for participants aged 15 and younger ensuring that all ethical guidelines were rigorously followed. Participants could opt out from the study at any stage. Participants were not compensated for their participation by the independent non-profit foundation Stiftung Gesundheitswissen and only anonymized data was provided to the Stiftung Gesundheitswissen.

To ensure transparency, we would like to emphasize that this study is part of a larger research project which collected data on health literacy, eating disorders, depression, social media usage, climate change distress and impairment, health and health behaviors in addition to sociodemographic information. For economic reasons, sample recruitment and data collection for different research questions and objectives were combined. Results are reported in separate publications (e.g., König et al., 2024b; König et al., 2024a) in order to provide a focused and detailed analysis and discussion for each research question.

### 2.2 Survey methodology and data acquisition

Data collection was carried out by two market research institutes. Data for the adult sample (≥ 16 years) was collected by forsa (Gesellschaft für Sozialforschung und statistische Analysen mbH) in December 2023 using the forsa.omninet online panel. The panel has around 100,000 participants and is representative of the German-speaking population with Internet access in Germany. Panelists are recruited offline via telephone on the basis of random samples. New panelists are recruited every month, and the composition of the panel is continuously monitored on the basis of key characteristics (e.g., region, age, gender) and recruitment is adjusted accordingly. For the present investigation, a random representative sample was drawn from the panelists 16 years and older. Data collection was carried out online with computer-assisted web interviews. A total of 3,011 participants provided complete questionnaires. Survey weights were calculated by forsa using an iterative proportional fitting approach according to the following weight variables and combinations: (a) gender × age (in the groups 16–29 years, 30–45 years, 46–64 years, ≥ 65 years) × region (West Germany and Berlin, East Germany) and (b) federal state. The weighting was based on the population census of the German Federal Statistical Office (as by 31.12.2021).

Data for the adolescent sample was collected by GIM (Gesellschaft für innovative Marktforschung mBH). The study population consisted of pupils aged 12 to 17 from Germany. Data collection was carried out with a mixed-mode approach using computer-assisted web interviews (about 2/3 of all interviews) via an online-access panel and computer-assisted face-to-face interviews (about 1/3 of all interviews). The sample was based on quotas for the factors age (12–13 years, 14–15 years and 16–17 years), gender, type of the currently attended school and federal state. A total of 1,021 complete questionnaires were obtained. Data collection was carried out in November and December 2023. Survey weights were calculated in an iterative procedure according to the following weight variables and combinations: age × gender, education and federal state. Quotas and weighting were based on data from the Federal Statistical Office and the ma Radio survey which in turn is based on the most recent Mikrozensus.

### 2.3 Measures

#### 2.3.1 Sociodemographic information

Basic sociodemographic information including gender (male, female, diverse [in adolescents only]) and age were obtained. Participants from the adult sample were categorized into four age groups (16–29 years, 30–45 years, 46–64 years, ≥ 65 years) and participants from the adolescent sample were categorized into three age groups (12–13 years, 14–15 years, 16–17 years).

In the adult sample, levels of education were obtained by asking participants to indicate their highest level of formal education and grouping them into three categories: low (equivalent to no formal education or basic secondary school; ohne Haupt-/Volksschulabschluss, Haupt-/Volksschulabschluss), middle (equivalent to intermediate secondary school; Mittlere Reife, Realschulabschluss, Fachschulreife, Abschluss der Polytechnischen Oberschule, Fachhochschulreife, Abschluss einer Fachoberschule) and high formal level of education (equivalent to most advanced secondary school, e.g., grammar schools to obtain a general or specialized university entrance qualification, or university degree; Abitur, allgemeine oder fachgebundene Hochschulreife, Fach-/Hochschulstudium). Adolescents were asked for the type of their currently attended school and divided into two levels of education: high (equivalent to most advanced secondary school, e.g., grammar schools, where a general university entrance qualification can be obtained; Gymnasium) and low (all other school types).

Subjective social status of the adult participants was assessed with the German version of the MacArthur scale (Hoebel et al., 2015; Adler et al., 2000). The scale represents subjective social status with a ladder metaphor, with rung 10 at the top symbolizing highest social status and rung 1 at the bottom denoting lowest social status. Participants are asked to identify the rung that best represents their position. Three categories of subjective social status were determined, i.e., low subjective social status (scores 1–4), middle subjective social status (scores 5–7) and high subjective social status (scores 8–10). In the adolescent sample, social status was assessed using the German version of the Revised Family Affluence Scale (FAS III; Torsheim et al., 2016; University Medicine Greifswald 12.07., 2024). The scale consists of six items asking the respondents about their family’s standard of living, e.g., if they have their own bedroom. A sum score is calculated by adding up answers across items, resulting in a range from 0 to 14. In this study, participants were divided into three categories, e.g., low (bottom 20%, scores 0–5), middle (middle 60%, scores 6–9), and high (top 20%, scores 10–14) social status (Moor et al., 2024; Corell et al., 2021).

#### 2.3.2 PHQ-9

Depressive symptoms were assessed with the German version of the depression module of the Patient Health Questionnaire (PHQ-9; Kroenke et al., 2001; Spitzer et al., 1999; Gräfe et al., 2004). The questionnaire is based on the diagnostic criteria for major depressive disorder from the DSM-IV. Each item represents a depressive symptom, and participants are asked to indicate how often in the past two weeks they were bothered by the symptom. Responses are assessed using a 4-point scale (0 = “not at all,” 1 = “several days,” 2 = “more than half of the days” or 3 = “nearly every day”). An overall score is calculated by summing up item responses. The overall score serves as severity measure with a range from 0 to 27. In addition, depressive categories have been defined according to the overall score, i.e., minimal (score 0–4), mild (score 5–9), moderate (score 10–14), moderately severe (score 15–19) and severe depressive symptom severity (score 20–27; Kroenke et al., 2001). A cut-off of ≥10 on the total score has been recommended to detect major depression maximizing both sensitivity and specificity (Kroenke et al., 2001; Levis et al., 2019). In addition, a diagnostic algorithm criterion has been developed (Spitzer et al., 1999; He et al., 2020), which requires 5 or more items to be present for more than half of the days or nearly every day, with at least one being depressed mood or anhedonia. Item 9 (suicidal ideation) is already met if the symptom has been present for at least several days. Analyses for both the cut-off and the diagnostic algorithm criteria are reported here. The PHQ-9 questionnaire has been widely validated in both adolescents and adults (Spitzer et al., 1999; Martin et al., 2006; Kroenke et al., 2001; Gräfe et al., 2004; Richardson et al., 2010; Fonseca-Pedrero et al., 2023).

#### 2.3.3 HLS-EU-Q16

The German translation of the short version of the European Health Literacy Survey instrument (HLS-EU-Q16) was employed to assess health literacy (Jordan and Hoebel, 2015; Pelikan et al., 2014; Sørensen et al., 2013). The 16 items refer to the domains accessing, understanding, appraising and applying information concerning healthcare, disease prevention and health promotion (Sørensen et al., 2013). Participants are asked to indicate perceived difficulty in these areas on a 4-point Likert scale (“very easy,” “fairly easy,” “fairly difficult,” “very difficult”). To obtain an overall score, item responses are first dichotomized (e.g., 1 = “fairly easy” and “very easy,” 0 = “fairly difficult” and “very difficult”). The final sum score is then calculated by adding up responses across the dichotomized items. Participants can be grouped according to their overall score, e.g., those with inadequate or problematic health literacy (scores 0–12) and those with adequate health literacy (scores 13–16; Pelikan et al., 2019).

### 2.4 Statistical analyses

All statistical analyses were conducted with the statistical software SPSS (version 29.0.2.0, IBM). All inferential statistical analyses are reported for the weighted data (see 2.2 for details on the weighting procedures). Participants were excluded from analyses of all measures for which they had missing data in at least one item. Internal consistencies were calculated using Cronbach’s *α*. To test whether there are significant differences between the sociodemographic and psychological factor levels in terms of PHQ-9 depressive symptom severity, *t*-tests (for the dichotomous factors including gender, education [in adolescents] and health literacy) and univariate analyses of variance (ANOVAs; for the factors with multiple levels including age categories, social status and education [in adults]). For the *t*-tests, Cohen’s *d* was calculated as a measure of effect size. If the Levene’s test for equality of variances indicated that variances were not homogeneous, degrees of freedom were adjusted accordingly. For the ANOVAs, η^2^_p_ was used as a measure of effect size. Significant ANOVA main effects were followed up with post-hoc *t*-tests for each pair of the factor levels. The post-hoc *t*-tests were tested for significance with a Bonferroni-corrected α-level. Uncorrected *p*-values are reported. In addition, χ^2^-tests of independence were calculated to analyze whether the proportions of individuals identified as likely having depression by the PHQ-9 criteria were independent of the categorial sociodemographic and psychological factors studied here (i.e., gender, age categories, social status, level of education, health literacy). Effect sizes for these tests are reported as Cramer’s *V*. Pearson correlation coefficients (covariance of the two variables divided by the product of their standard deviations) were calculated to assess the association between depressive symptom severity and health literacy. Correlation coefficients were not adjusted for additional variables.

## 3 Results

### 3.1 Adults

#### 3.1.1 Sample characteristics

The adult sample consisted of *N* = 3,011 individuals. The sociodemographic characteristics (i.e., gender, age, level of education, social status) and health literacy categories of the adult sample before (unweighted) and after (weighted) the weighting procedure can be found in Table 1.

**Table 1:** Sample characteristics of the weighted and unweighted sample of adults.

Variable	Unweighted sample, *N* (%)	Weighted sample, *N* (%)
Gender		
Male	1,483 (49.3%)	1,475 (49.0%)
Female	1,528 (50.7%)	1,536 (51.0%)
Age		
16–29 years	535 (17.8%)	531 (17.6%)
30–45 years	658 (21.9%)	721 (24.0%)
46–64 years	946 (31.4%)	976 (32.4%)
≥ 65 years	872 (29.0%)	783 (26.0%)
Level of education		
Low	768 (25.5%)	691 (23.0%)
Middle	1,110 (36.9%)	1,147 (38.1%)
High	1,130 (37.5%)	1,170 (38.8%)
Missing values	3 (0.1%)	3 (0.1%)
Social status		
Low	418 (13.9%)	426 (14.2%)
Middle	1,992 (66.2%)	1,988 (66.0%)
High	597 (19.8%)	593 (19.7%)
Missing values	4 (0.1%)	4 (0.1%)
Health literacy		
Inadequate/problematic	1,192 (39.6%)	1,201 (39.9%)
Adequate	1,790 (59.4%)	1,784 (59.2%)
Missing values	29 (1.0%)	26 (0.9%)

#### 3.1.2 Quality of measures

In the adult sample, internal consistency of the PHQ-9 questionnaire was *α* = 0.87, internal consistency of the HLS-EU-Q16 questionnaire was *α* = 0.82.

#### 3.1.3 PHQ-9 and its associations with sociodemographic factors in adults

The absolute numbers and proportions of individuals for each level of depressive symptom severity and the absolute numbers and proportions of individuals meeting the cut-off ≥10 and diagnostic algorithm depression criteria are shown in Tables 2, 3, respectively. Descriptive results (means and standard deviations) for all sociodemographic factor levels and the health literacy categories are shown in Table 4. Details on responses to each PHQ-9 item are in Supplementary Table S1.

**Table 2:** Absolute numbers and proportions of individuals for each level of depressive symptom severity according to the PHQ-9 sum score in the adult and adolescent samples.

	No or minimal (score of 0–4)	Mild (score of 5–9)	Moderate (score of 10–14)	Moderately severe (score of 15–19)	Severe (score of 20–27)	Missing data in at least one PHQ-9 item
Adults	*N* = 1,604 (53.3%)	*N* = 904 (30.0%)	*N* = 334 (11.1%)	*N* = 109 (3.6%)	*N* = 53 (1.7%)	*N* = 7 (0.2%)
Adolescents	*N* = 598 (58.5%)	*N* = 235 (23.0%)	*N* = 106 (10.4%)	*N* = 46 (4.5%)	*N* = 36 (3.6%)	*N* = 0 (0.0%)

**Table 3:** Absolute numbers and proportions of individuals fulfilling and not fulfilling the depression criteria according to the cut-off of ≥10 and the diagnostic algorithm in the adult and adolescent samples.

	Cut-off of sum score ≥ 10		Diagnostic algorithm cut-off		Missing data in at least one PHQ-9 item
	Depression	No depression	Depression	No depression	
Adults	*N* = 497 (16.5%)	*N* = 2,508 (83.3%)	*N* = 217 (7.2%)	*N* = 2,788 (92.6%)	*N* = 7 (0.2%)
Adolescents	*N* = 188 (18.4%)	*N* = 833 (81.6%)	*N* = 100 (9.8%)	*N* = 921 (90.2%)	*N* = 0 (0.0%)

**Table 4:** PHQ-9 means and standard deviations for the sociodemographic factors and the health literacy categories in the adult sample.

	M	SD
Entire sample		
	5.38	4.68
Gender		
Male	4.83	4.39
Female	5.91	4.89
Age		
16–29 years	7.04	4.95
30–45 years	6.12	4.79
46–64 years	5.10	4.68
≥ 65 years	3.93	3.85
Level of education		
Low	4.94	4.65
Middle	5.40	4.74
High	5.62	4.62
Social status		
Low	8.00	5.60
Middle	5.27	4.43
High	3.87	3.95
Health literacy		
Inadequate/problematic	6.97	5.26
Adequate	4.32	3.91

Women reported significantly higher PHQ-9 symptom severity than men, *t*(2988.89) = −6.34, *p* < 0.001, *d* = −0.23. The χ^2^-tests of independence between each of the two dichotomous depression categories (sum score ≥ 10 and diagnostic algorithm) and gender were significant (details in Tables 5, 6).

**Table 5:** Proportions of adults likely and not likely to be depressed according to the PHQ-9 sum score ≥ 10 criterion across sociodemographic factors and health literacy categories.

	Proportion of individuals likely to be depressed, PHQ-9 sum score ≥ 10	Proportion of individuals not likely to be depressed, PHQ-9 sum score < 10	*χ* ^2^-test of independence
Gender			
Male	12.9%	87.1%	χ^2^ (1, *N* = 3,003) = 26.94, *p <* 0.001, *V* = 0.10
Female	20.0%	80.0%	
Age			
16–29 years	26.8%	73.2%	χ^2^ (3, *N* = 3,004) = 82.41, *p <* 0.001, *V* = 0.17
30–45 years	19.9%	80.1%	
46–64 years	14.7%	85.3%	
≥ 65 years	8.8%	91.2%	
Level of education			
Low	14.5%	85.5%	χ^2^ (2, *N* = 3,003) = 2.78, *p =* 0.249, *V* = 0.03
Middle	16.9%	83.1%	
High	17.4%	82.6%	
Social status			
Low	33.3%	66.7%	χ^2^ (2, *N* = 3,001) = 118.16, *p <* 0.001, *V* = 0.20
Middle	15.5%	84.5%	
High	8.1%	91.9%	
Health literacy			
Inadequate/problematic	26.8%	73.3%	χ^2^ (1, *N* = 2,982) = 150.68, *p <* 0.001, *V* = 0.23
Adequate	9.7%	90.3%	

**Table 6:** Proportions of adults likely and not likely to be depressed according to the PHQ-9 diagnostic algorithm criterion across sociodemographic factors and health literacy categories.

	Proportion of individuals likely to be depressed, meet diagnostic algorithm criterion	Proportion of individuals not likely to be depressed, do not meet diagnostic algorithm criterion	χ ^2^-test of independence
Gender			
Male	5.6%	94.4%	χ^2^ (1, *N* = 3,005) = 10.72, *p =* 0.001, *V* = 0.06
Female	8.7%	91.3%	
Age			
16–29 years	13.4%	86.6%	χ^2^ (3, *N* = 3,005) = 50.96, *p <* 0.001, *V* = 0.13
30–45 years	8.3%	91.7%	
46–64 years	6.2%	93.8%	
≥ 65 years	3.3%	96.7%	
Level of education			
Low	7.5%	92.5%	χ^2^ (2, *N* = 3,001) = 0.42, *p =* 0.812, *V* = 0.01
Middle	6.8%	93.2%	
High	7.4%	92.6%	
Social status			
Low	17.0%	83.0%	χ^2^ (2, *N* = 3,002) = 75.85, *p <* 0.001, *V* = 0.16
Middle	6.3%	93.7%	
High	3.4%	96.6%	
Health literacy			
Inadequate/problematic	12.2%	87.8%	χ^2^ (1, *N* = 2,982) = 72.44, *p <* 0.001, *V* = 0.16
Adequate	3.9%	96.1%	

There was a significant effect of age on PHQ-9 symptom severity, *F*(3,3000) = 57.49, *p* < 0.001, η^2^_p_ = 0.054. Descriptively, the sum scores decreased with increasing age. Post-hoc Bonferroni-corrected *t*-tests revealed significant differences between all four groups. The 16–29-year-olds reported highest symptom severity and differed significantly from the 30–45-year-olds, *t*(1247) = 3.29, *p* = 0.001, *d* = 0.19, from the 46–64-year-olds *t*(1035.57) = 7.39, *p* < 0.001, *d* = 0.41, and from the ≥65-year-olds, *t*(944.29) = 12.18, *p* < 0.001, *d* = 0.72. There were also significant differences between the 30–45-year-olds and the 46–64 year-olds, *t*(1689) = 4.41, *p* < 0.001, *d* = 0.22, the 30–45-year-olds and ≥ 65-year-olds, *t*(1379.26) = 9.74, *p* < 0.001, *d* = 0.51, and between the 46–64-year-olds and ≥ 65-year-olds, *t*(1751.90) = 5.75, *p* < 0.001, *d* = 0.27. The χ^2^-tests of independence between each of the two dichotomous depression categories and the four age groups were significant (details in Tables 5, 6).

There was a significant effect of level of education on PHQ-9 symptom severity, *F*(2,2999) = 4.59, *p* = 0.010, η^2^_p_ = 0.003. Post-hoc Bonferroni-corrected *t*-tests revealed significant differences only between the low and high educational levels *t*(1856) = −3.05, *p* = 0.002, *d* = −0.15, but not between the low and middle educational levels, *t*(1833) = −2.04, *p* = 0.042, *d* = −0.10 (not significant after Bonferroni-correction), and between the middle and high educational levels, *t*(2310) = −1.11, *p* = 0.268, *d* = −0.05. Symptom severity was highest in those with high educational levels and lowest in those with low educational levels, with those with middle educational levels scoring in between the two other groups. The χ^2^-tests of independence between each of the two dichotomous depression categories and the three levels of education were not significant (details in Tables 5, 6).

There was a significant effect of subjective social status on PHQ-9 symptom severity, *F*(2,2998) = 104.53, *p* < 0.001, η^2^_p_ = 0.065. Descriptively, the sum scores decreased with increasing subjective social status. Post-hoc Bonferroni-corrected *t*-tests revealed significant differences between all three groups (low vs. middle subjective social status: *t*(542.24) = 9.42, *p* < 0.001, *d* = 0.59, low vs. high subjective social status: *t*(713.21) = 13.05, *p* < 0.001, *d* = 0.88, middle vs. high subjective social status: *t*(1076.81) = 7.37, *p* < 0.001, *d* = 0.32). Symptom severity was highest in those with low subjective social status and lowest in those with high subjective social status, with those with middle subjective social status scoring in between the two other groups. The χ^2^-tests of independence between each of the two dichotomous depression categories and the three subjective social status groups were significant (details in Tables 5, 6).

#### 3.1.4 PHQ-9 and its associations with health literacy

There was a significant negative correlation between the PHQ-9 depression sum score and health literacy measured with the HLS-EU-Q16, *r* = −0.33, *p* < 0.001.

For the categorial variables, individuals with inadequate and problematic health literacy reported significantly higher PHQ-9 symptom severity than individuals with adequate health literacy, *t*(2066.74) = 14.90, *p* < 0.001, *d* = 0.59. The χ^2^-tests of independence between each of the two dichotomous depression categories and the two health literacy groups were significant (details in Tables 5, 6).

### 3.2 Adolescents

#### 3.2.1 Sample characteristics

The adolescent sample consisted of *N* = 1,021 individuals. The sociodemographic characteristics (i.e., gender, age, level of education, social status) and health literacy categories of the adolescent sample before (unweighted) and after (weighted) the weighting procedure can be found in Table 7.

**Table 7:** Sample characteristics of the weighted and unweighted sample of adolescents.

Variable	Unweighted sample, *N* (%)	Weighted sample, *N* (%)
Gender		
Male	514 (50.3%)	527 (51.6%)
Female	504 (49.4%)	492 (48.2%)
Diverse	3 (0.3%)	3 (0.3%)
Age		
12–13 years	340 (33.3%)	339 (33.2%)
14–15 years	346 (33.9%)	343 (33.6%)
16–17 years	335 (32.8%)	339 (33.2%)
Level of education		
Low	603 (59.1%)	603 (59.1%)
High	418 (40.9%)	418 (40.9%)
Social status		
Low	115 (11.3%)	114 (11.2%)
Middle	668 (65.4%)	666 (65.2%)
High	238 (23.3%)	241 (23.6%)
Health literacy		
Inadequate/problematic	548 (53.7%)	544 (53.3%)
Adequate	473 (46.3%)	477 (46.7%)

#### 3.2.2 Quality of measures

In the adolescent sample, internal consistency of the PHQ-9 questionnaire was *α* = 0.93, internal consistency of the HLS-EU-Q16 questionnaire was *α* = 0.88.

#### 3.2.3 PHQ-9 and its associations with sociodemographic factors in adolescents

The absolute numbers and proportions of adolescents for each level of depressive symptom severity and the absolute numbers and proportions of individuals meeting the cut-off ≥10 and diagnostic algorithm depression criteria are shown in Tables 2, 3, respectively. Descriptive results (means and standard deviations) for all sociodemographic factor levels and the health literacy categories are shown in Table 8. Details on responses to each PHQ-9 item are in Supplementary Table S2.

**Table 8:** PHQ-9 means and standard deviations for the sociodemographic factors and the health literacy categories in the adolescent sample.

	M	SD
Entire sample		
	5.01	5.64
Gender		
Male	4.36	5.29
Female	5.66	5.89
Age		
12–13 years	4.02	4.31
14–15 years	4.88	5.55
16–17 years	6.14	6.64
Level of education		
Low	4.90	5.40
High	5.19	5.97
Social status		
Low	6.06	6.65
Middle	4.62	5.19
High	5.60	6.20
Health literacy		
Inadequate/problematic	6.48	6.14
Adequate	3.34	4.46

Individuals who identify as diverse were excluded from the following analyses concerning gender due to low cell frequencies. Girls reported significantly higher PHQ-9 symptom severity than boys, *t*(985.72) = −3.71, *p* < 0.001, *d* = −0.23. The χ^2^-test of independence between the sum score ≥ 10 depression categories and gender was significant (details in Table 9) but the χ^2^-test of independence between the diagnostic algorithm categories and gender was not significant (details in Table 10).

**Table 9:** Proportions of adolescents likely and not likely to be depressed according to the PHQ-9 sum score ≥ 10 criterion across sociodemographic factors and health literacy categories.

	Proportion of individuals likely to be depressed, PHQ-9 sum score ≥ 10	Proportion of individuals not likely to be depressed, PHQ-9 sum score < 10	χ ^2^-test of independence
Gender			
Male	15.4%	84.6%	χ^2^ (1, *N* = 1,018) = 5.77, *p =* 0.016, *V* = 0.08
Female	21.2%	78.8%	
Age			
12–13 years	12.4%	87.6%	χ^2^ (2, *N* = 1,022) = 18.17, *p <* 0.001, *V* = 0.13
14–15 years	18.0%	82.0%	
16–17 years	25.1%	74.9%	
Level of education			
Low	18.7%	81.3%	χ^2^ (1, *N* = 1,021) = 0.10, *p* = 0.747, *V* = 0.01
High	17.9%	82.1%	
Social status			
Low	25.4%	74.6%	χ^2^ (2, *N* = 1,021) = 8.56, *p* = 0.014, *V* = 0.09
Middle	15.9%	84.1%	
High	22.0%	78.0%	
Health literacy			
Inadequate/problematic	27.9%	72.1%	χ^2^ (1, *N* = 1,021) = 69.88, *p <* 0.001, *V* = 0.26
Adequate	7.6%	92.4%	

**Table 10:** Proportions of adolescents likely and not likely to be depressed according to the PHQ-9 diagnostic algorithm criterion across sociodemographic factors and health literacy categories.

	Proportion of individuals likely to be depressed, meet diagnostic algorithm criterion	Proportion of individuals not likely to be depressed, do not meet diagnostic algorithm criterion	χ ^2^-test of independence
Gender			
Male	8.0%	92.0%	χ^2^ (1, *N* = 1,019) = 3.79, *p =* 0.051, *V* = 0.06
Female	11.6%	88.4%	
Age			
12–13 years	4.1%	95.9%	χ^2^ (2, *N* = 1,022) = 22.87, *p <* 0.001, *V* = 0.15
14–15 years	10.5%	89.5%	
16–17 years	15.0%	85.0%	
Level of education			
Low	10.0%	90.0%	χ^2^ (1, *N* = 1,021) = 0.04, *p* = 0.840, *V* = 0.01
High	9.6%	90.4%	
Social status			
Low	17.5%	82.5%	χ^2^ (2, *N* = 1,021) = 12.56, *p* = 0.002, *V* = 0.11
Middle	7.7%	92.3%	
High	12.0%	88.0%	
Health literacy			
Inadequate/problematic	14.5%	85.5%	χ^2^ (1, *N* = 1,020) = 29.35, *p <* 0.001, *V* = 0.17
Adequate	4.4%	95.6%	

There was a significant effect of age on PHQ-9 symptom severity, *F*(2,1017) = 12.42, *p* < 0.001, η^2^*_p_* = 0.024. Descriptively, the sum scores increased with increasing age: scores were lowest for the youngest age group, followed by the 14–15-year-olds. The 16–17-year-olds reported highest symptom severity. Post-hoc Bonferroni-corrected *t*-tests revealed significant differences between the 12–13-year-olds and the 16–17-year-olds, *t*(579.30) = −4.94, *p* < 0.001, *d* = −0.38, and between the 14–15-year-olds and the 16–17-year-olds, *t*(656.12) = −2.70, *p* = 0.007, *d* = −0.21, but not between the two younger groups, *t*(644.88) = −2.26, *p* = 0.024, *d* = −0.17. The χ^2^-tests of independence between each of the two dichotomous depression categories and the four age groups were significant (details in Tables 9, 10).

There was no significant effect of level of education on PHQ-9 symptom severity, *t*(1019) = −0.81, *p* = 0.419, *d* = −0.05. Descriptively, symptom severity was higher in high vs. low educational levels. The χ^2^-tests of independence between each of the two dichotomous depression categories and the two levels of education were also not significant (details in Tables 9, 10).

There was a significant effect of subjective social status on PHQ-9 symptom severity, *F*(2,1017) = 4.91, *p* = 0.008, η^2^_p_ = 0.010. Post-hoc Bonferroni-corrected *t*-tests revealed no significant differences for any other pair of comparison: low and middle social status groups, *t*(137.52) = 2.19, *p* = 0.030, *d* = 0.26, low vs. high social status, *t*(353) = 0.63, *p* = 0.531, *d* = 0.07, middle vs. high social status: *t*(369.26) = −2.20, *p* = 0.029, *d* = −0.18. Descriptively, the sum scores were lowest for the middle social status group, followed by the high social status group and the low social status group. The χ^2^-tests of independence between each of the two dichotomous depression categories and the three subjective social status groups were significant (details in Tables 9, 10).

#### 3.2.4 PHQ-9 and its associations with health literacy

There was a significant negative correlation between the PHQ-9 depression sum score and health literacy measured with the HLS-EU-Q16, *r* = −0.30, *p* < 0.001.

For the categorial variables, individuals with inadequate and problematic health literacy reported significantly higher PHQ-9 symptom severity than individuals with adequate health literacy, *t*(986.96) = 9.44, *p* < 0.001, *d* = 0.58. The χ^2^-tests of independence between each of the two dichotomous depression categories and the two health literacy groups were significant (details in Tables 9, 10).

## 4 Discussion

The current study investigated the associations between depressive symptoms, health literacy and additional sociodemographic factors in two large sample of adolescents and adults representative of the German-speaking population in Germany.

### 4.1 Depressive symptoms within the population

Overall, rates of depression were high in both samples (16.5% in adults and 18.4% in adolescents) when measured with the sum score ≥ 10 cut-off criterion and substantially lower when assessed with the diagnostic algorithm criterion (7.2% in adults and 9.8% in adolescents). With both approaches, rates are substantially higher than those from previous investigations in Germany (Maske et al., 2015; Kocalevent et al., 2013). In addition, the mean sum score was also higher in our samples compared to some (Hinz et al., 2016) but not all earlier findings (Shevlin et al., 2022; Stocker et al., 2021). This finding may be interpreted as an increase in depression rates and severity in the past years in Germany, consistent with an overall rise worldwide (Liu et al., 2020). However, to our knowledge, this study was the first to comprehensively use the PHQ-9 questionnaire in representative online samples in Germany. It cannot be ruled out that responses are influenced by the interview mode, resulting in higher depression values when measured online vs. in a face-to-face or telephone interview (Zhang et al., 2017). Higher rates in Germany compared to other European countries have been found before and may be due to higher rates of mild but not moderate or severe depression severity (Hapke et al., 2019). Importantly, differences occur particularly in younger age groups, consistent with our findings of elevated depression rates in late adolescence (Hapke et al., 2019). Differences in the observed depression rates between the diagnostic algorithm and the total score ≥ 10 cut-off criterion have been reported before (Levis et al., 2020). Diverging results may be due to different validation objectives for each approach. The total score cut-off of ≥10 has relatively high sensitivity and specificity (Levis et al., 2019), whereas the diagnostic algorithm yields very high specificity but lower sensitivity (He et al., 2020). Consequently, the cut-off of ≥10 has been found to overestimate depression prevalences (Levis et al., 2020), while the diagnostic algorithm criterion might miss true cases due to lower sensitivity (Levis et al., 2020). However, both approaches are valid to give estimates of the number of individuals affected by depressive symptoms and can be interpreted for different study purposes.

In line with previous research using the PHQ-9 and clinical interviews, we found significant gender differences pointing to higher rates of depression and higher depression severity in female compared to male participants in both samples (Maske et al., 2015; Kocalevent et al., 2013; Jacobi et al., 2014; Shorey et al., 2022).

In the adult sample, depression rates decreased with increasing age but increased with increasing age in the adolescent sample. The decline in older adults is in contrast with some (Kocalevent et al., 2013; Bianchi et al., 2022) but not all previous investigations using the PHQ-9 (Stocker et al., 2021). Importantly, lower depression rates in older vs. younger adults have also been found in epidemiological studies using diagnostic interviews (Jacobi et al., 2014), in line with our finding in the present study. High and rising depression rates in adolescents, similar to those found in other studies (Andreas et al., 2017; Shorey et al., 2022), are worrying because adolescence represents a sensitive developmental phase, increasing the risk of persisting mental health problems later in life (Lee et al., 2014; Johnson et al., 2018). Therefore, adolescents might particularly benefit from prevention programs.

In the adult sample, level of education influenced depression symptom severity as a continuous variable, with highest severity in individuals with high educational levels and lowest severity in those with low levels of education. When depression was interpreted as a categorial variable, there were no significant associations with levels of education. Likewise, in adolescents, levels of education were not associated with depression severity or categories. These findings are in contrast to earlier studies pointing to increased levels of depression in individuals with lower levels of education (Kocalevent et al., 2013; Hapke et al., 2022; Tüzün et al., 2021; Tassone et al., 2024).

There was a marked effect of subjective social status on depression severity and categories in adults. Lower subjective social status was associated with higher depression severity and rates. In adolescents, however, this pattern of results was not replicated. Instead, depression severity was lowest in individuals with middle social status compared to low and high social status. Differences between the two samples might be accounted for by the different instruments used to assess social status, with the FAS-III used in adolescents more strongly reflecting the economic situation of the family and the MAS in adults focusing on perceived status in comparison to other members of society (Torsheim et al., 2016; Adler et al., 2000). In past investigations – as in our adult sample – lower social status or rank has been associated with higher rates of depressive symptoms (Wetherall et al., 2019). Importantly, the direction of the relationship between the two constructs is subject of ongoing debate but perceived low social rank might be a factor in depression etiology (Wetherall et al., 2019).

### 4.2 Health literacy and depression

Negative correlations between depressive symptom severity and health literacy were observed in both samples. Similar results were found for the categorial variables: adolescents and adults with inadequate and problematic health literacy were approximately three times more likely to be depressed than those with adequate health literacy. These results are directly in line with earlier studies on the association of health literacy and depressive symptoms (Mo et al., 2023; Gazmararian et al., 2000; Lincoln et al., 2006; Guo et al., 2023; Smith and Moore, 2012). Although the direction of the association cannot be determined with our cross-sectional design, addressing both depressive symptoms and health literacy in prevention programs is a promising avenue to improve overall (mental) health (Smith and Moore, 2012). More specifically, our findings suggest that improving health literacy may serve as an effective preventive measure against depression, particularly in vulnerable populations. Positive effects of health literacy interventions on various health outcomes have been delineated in the past (Mallia et al., 2020; Walters et al., 2020). Critically, there is first evidence to suggest positive effects of health literacy interventions on depressive symptomology (Uemura et al., 2021). Preventions programs focusing on enhancing health literacy could help to promote health care utilization, emergency response, patient-provider relationships and self-care in order to improve health outcomes including depression (Paasche-Orlow and Wolf, 2007; Berkman et al., 2011; Smith and Moore, 2012; Uemura et al., 2021; Diotaiuti et al., 2021). In addition, particularly in adolescents, focused interventions might help to empower individuals to make independent and informed health-decisions with positive effects on their current and long-term health (Fleary et al., 2018). However, more high-quality research in this area is needed, including randomized controlled trials to evaluate the efficacy of prevention programs for different risk groups.

### 4.3 Strengths and limitations

To our knowledge, this was the first study to comprehensively assess depressive symptoms and health literacy in two large and representative samples of adolescents and adults in Germany. High Cronbach’s *α* internal consistencies of the PHQ-9 and HLS-EU-16 questionnaires highlight the overall reliability of the measures (Kroenke et al., 2010; Jordan and Hoebel, 2015).

However, there are also some limitations to this study. First, the adult sample is only representative of the German-speaking population with internet access in Germany. Therefore, the study findings cannot be generalized to individuals with insufficient knowledge of the German language and to those who do not have internet access or do not use the internet. As a result, certain segments of the population at particular risk for low health literacy may have been overlooked. Second, the study employed a cross-sectional design. Consequently, causal relationships between the investigated constructs cannot be inferred (Wang and Cheng, 2020). Future studies with longitudinal and experimental designs are strongly recommended for more comprehensive analyses and to allow causal inferences. Third, the HLS-EU-Q16 questionnaire might not be an optimal measure of health literacy in adolescents due to a lack of familiarity and relevance of some of the items, as well as difficulties in abstracting (Domanska et al., 2018). This shortcoming may lead to an overestimation of adolescents with inadequate or problematic health literacy (Domanska et al., 2018). Indeed, rates of individuals with inadequate or problematic health literacy were higher among adolescents compared to adults in our data. Fourth, as noted above, although the PHQ-9 has overall good psychometric properties, it may lead to a overestimation of the prevalence of depressive disorders in the population (Levis et al., 2020), which is why the rates of positive PHQ-9 screens in the present study should not be mistaken as accurate estimates of prevalences of depression in Germany, which are usually lower (e.g., Jacobi et al., 2014). Lastly, this study was explorative in nature, which is why only univariate analyses were conducted. These analyses cannot account for shared variance and interactions between the investigated factors. This limitation should be addressed in the future with more in-depth analyses, including multivariate models, to explore the separate and combined effects of sociodemographic factors and health literacy on depressive symptom levels.

### 4.4 Summary and conclusion

The purpose of the present study was to explore the relationship between depressive symptoms and health literacy in addition to sociodemographic factors in two large samples of adolescents and adults. Rates of depression and severity of depressive symptoms were higher in female than male adolescents and adults. Both depressive symptom severity and depression rates increased with increasing age in adolescents and decreased with increasing age in adults, indicating that adolescence and early adulthood are the age groups with the highest depressive symptom severity and rates. Higher levels of education were associated with higher depressive symptom severity and rates in adults but not in adolescents, where no associations between depressive symptoms and levels of education could be delineated. In adults, individuals with higher social status had lower depressive symptoms severity and rates, with a less clear picture in adolescents. Negative correlations between depressive symptom severity and health literacy highlight the importance of health literacy as a potential factor in depression prevention strategies. Collectively, our results highlight the need for targeted prevention programs in individuals at risk for developing depressive symptoms. Future research should aim to explore the effectiveness of health literacy interventions in diverse populations to assess their potential in reducing depressive symptoms longitudinally.

## Data Availability

The datasets presented in this article are not readily available because the datasets generated and analyzed during this study are the property of the independent, nonprofit foundation Stiftung Gesundheitswissen and are available on reasonable request. Requests to access the datasets should be directed to lars.koenig@stiftung-gesundheitswissen.de.

## References

[ref1] Adler N. E. Epel E. S. Castellazzo G. Ickovics J. R. (2000). Relationship of subjective and objective social status with psychological and physiological functioning: preliminary data in healthy white women. Health Psychol. 19, 586–592. doi: 10.1037/0278-6133.19.6.586, PMID: 11129362

[ref2] Alonso J. Angermeyer M. C. Bernert S. Bruffaerts R. Brugha T. S. Bryson H.. (2004). Disability and quality of life impact of mental disorders in Europe: results from the European study of the epidemiology of mental disorders (ESEMeD) project. Acta Psychiatr. Scand. Suppl. 109, 38–46. doi: 10.1111/j.1600-0047.2004.00329.x, PMID: 15128386

[ref3] Andreas B. Jasmina B. Geir S. (2017). Depressive symptomatology among Norwegian adolescent boys and girls: the patient health Questionnaire-9 (PHQ-9) psychometric properties and correlates. Front. Psychol. 8:887. doi: 10.3389/fpsyg.2017.00887, PMID: 28642720 PMC5462997

[ref4] Andrews G. Issakidis C. Sanderson K. Corry J. Lapsley H. (2004). Utilising survey data to inform public policy: comparison of the cost-effectiveness of treatment of ten mental disorders. Br. J. Psychiatry 184, 526–533. doi: 10.1192/bjp.184.6.526, PMID: 15172947

[ref5] Barth J. Munder T. Gerger H. Nüesch E. Trelle S. Znoj H.. (2016). Comparative efficacy of seven psychotherapeutic interventions for patients with depression: a network Meta-analysis. Focus 14, 229–243. doi: 10.1176/appi.focus.140201, PMID: 31997951 PMC6519647

[ref6] Bellón J. Á. Conejo-Cerón S. Cortés-Abela C. Pena-Andreu J. M. García-Rodríguez A. Moreno-Peral P. (2019). Effectiveness of psychological and educational interventions for the prevention of depression in the workplace: a systematic review and meta-analysis. Scand. J. Work Environ. Health 45, 324–332. doi: 10.5271/sjweh.3791 30500058

[ref7] Bellón J. Á. Moreno-Peral P. Motrico E. Rodríguez-Morejón A. Fernández A. Serrano-Blanco A.. (2015). Effectiveness of psychological and, or educational interventions to prevent the onset of episodes of depression: a systematic review of systematic reviews and meta-analyses. Prev. Med. 76, S22–S32. doi: 10.1016/j.ypmed.2014.11.003 25445331

[ref8] Berkman N. D. Sheridan S. L. Donahue K. E. Halpern D. J. Crotty K. (2011). Low health literacy and health outcomes: an updated systematic review. Ann. Intern. Med. 155, 97–107. doi: 10.7326/0003-4819-155-2-201107190-00005, PMID: 21768583

[ref9] Bianchi R. Verkuilen J. Toker S. Schonfeld I. S. Gerber M. Brähler E.. (2022). Is the PHQ-9 a unidimensional measure of depression? A 58,272-participant study. Psychol. Assess. 34, 595–603. doi: 10.1037/pas0001124, PMID: 35357877

[ref10] Bromet E. Andrade L. H. Hwang I. Sampson N. A. Alonso J. Girolamo G.. (2011). Cross-national epidemiology of DSM-IV major depressive episode. BMC Med. 9:90. doi: 10.1186/1741-7015-9-90, PMID: 21791035 PMC3163615

[ref11] Bundesärztekammer (BÄK), Kassenärztliche Bundesvereinigung (KBV), Arbeitsgemeinschaft der Wissenschaftlichen Medizinischen Fachgesellschaften (AWMF). (2022). Nationale VersorgungsLeitlinie Unipolare Depression – Langfassung, Version 3.0: Konsultationsfassung. Available at: https://www.leitlinien.de/themen/depression

[ref12] Chan J. K. N. Correll C. U. Wong C. S. M. Chu R. S. T. Fung V. S. C. Wong G. H. S.. (2023). Life expectancy and years of potential life lost in people with mental disorders: a systematic review and meta-analysis. EClinicalMedicine 65:102294. doi: 10.1016/j.eclinm.2023.102294, PMID: 37965432 PMC10641487

[ref13] Chesney E. Goodwin G. M. Fazel S. (2014). Risks of all-cause and suicide mortality in mental disorders: a meta-review. World Psychiatry 13, 153–160. doi: 10.1002/wps.20128 24890068 PMC4102288

[ref14] Conejo-Cerón S. Moreno-Peral P. Rodríguez-Morejón A. Motrico E. Navas-Campaña D. Rigabert A.. (2017). Effectiveness of psychological and educational interventions to prevent depression in primary care: a systematic review and Meta-analysis. Ann. Fam. Med. 15, 262–271. doi: 10.1370/afm.2031, PMID: 28483893 PMC5422089

[ref15] Corell M. Chen Y. Friberg P. Petzold M. Löfstedt P. (2021). Does the family affluence scale reflect actual parental earned income, level of education and occupational status? A validation study using register data in Sweden. BMC Public Health 21:1995. doi: 10.1186/s12889-021-11968-2, PMID: 34732163 PMC8565642

[ref16] Cuijpers P. Noma H. Karyotaki E. Vinkers C. H. Cipriani A. Furukawa T. A. (2020). A network meta-analysis of the effects of psychotherapies, pharmacotherapies and their combination in the treatment of adult depression. World Psychiatry 19, 92–107. doi: 10.1002/wps.20701, PMID: 31922679 PMC6953550

[ref17] Cuijpers P. Quero S. Noma H. Ciharova M. Miguel C. Karyotaki E.. (2021). Psychotherapies for depression: a network meta-analysis covering efficacy, acceptability and long-term outcomes of all main treatment types. World Psychiatry 20, 283–293. doi: 10.1002/wps.20860, PMID: 34002502 PMC8129869

[ref18] Deady M. Choi I. Calvo R. A. Glozier N. Christensen H. Harvey S. B. (2017). eHealth interventions for the prevention of depression and anxiety in the general population: a systematic review and meta-analysis. BMC Psychiatry 17:310. doi: 10.1186/s12888-017-1473-1 28851342 PMC5576307

[ref19] Diotaiuti P. Valente G. Mancone S. (2021). Development and preliminary Italian validation of the emergency response and psychological adjustment scale. Front. Psychol. 12:687514. doi: 10.3389/fpsyg.2021.687514, PMID: 34421737 PMC8376143

[ref20] Domanska O. M. Firnges C. Bollweg T. M. Sørensen K. Holmberg C. Jordan S. (2018). Do adolescents understand the items of the European health literacy survey questionnaire (HLS-EU-Q47) - German version? Findings from cognitive interviews of the project "measurement of health literacy among adolescents" (MOHLAA) in Germany. Arch. Public Health 76:46. doi: 10.1186/s13690-018-0276-2, PMID: 30009022 PMC6040081

[ref21] Fleary S. A. Joseph P. Pappagianopoulos J. E. (2018). Adolescent health literacy and health behaviors: a systematic review. J. Adolesc. 62, 116–127. doi: 10.1016/j.adolescence.2017.11.010 29179126

[ref22] Flint J. Kendler K. S. (2014). The genetics of major depression. Neuron 81, 484–503. doi: 10.1016/j.neuron.2014.01.027, PMID: 24507187 PMC3919201

[ref23] Fonseca-Pedrero E. Díez-Gómez A. Pérez-Albéniz A. Al-Halabí S. Lucas-Molina B. Debbané M. (2023). Youth screening depression: validation of the patient health Questionnaire-9 (PHQ-9) in a representative sample of adolescents. Psychiatry Res. 328:115486. doi: 10.1016/j.psychres.2023.115486 37738682

[ref24] Gazmararian J. Baker D. Parker R. Blazer D. G. (2000). A multivariate analysis of factors associated with depression: evaluating the role of health literacy as a potential contributor. Arch. Intern. Med. 160, 3307–3314. doi: 10.1001/archinte.160.21.3307 11088094

[ref25] GBD 2019 Mental Disorders Collaborators (2022). Global, regional, and national burden of 12 mental disorders in 204 countries and territories, 1990-2019: a systematic analysis for the global burden of disease study 2019. Lancet Psychiatry 9, 137–150. doi: 10.1016/S2215-0366(21)00395-3, PMID: 35026139 PMC8776563

[ref26] Gräfe K. Zipfel S. Herzog W. Löwe B. (2004). Screening psychischer Störungen mit dem “Gesundheitsfragebogen für Patienten (PHQ-D)”. Diagnostica 50, 171–181. doi: 10.1026/0012-1924.50.4.171

[ref27] Guo C. Cui Y. Xia Z. Hu J. Xue Y. Huang X.. (2023). Association between health literacy, depressive symptoms, and suicide-related outcomes in adolescents: a longitudinal study. J. Affect. Disord. 327, 15–22. doi: 10.1016/j.jad.2023.01.054, PMID: 36707037

[ref28] Hapke U. Cohrdes C. Nübel J. (2019). Depressive symptoms in a European comparison – results from the European health interview survey (EHIS) 2. J. Health Monitor. 4, 57–65. doi: 10.25646/6227 PMC8734092 35146259

[ref29] Hapke U. Kersjes C. Hoebel J. Kuhnert R. Eicher S. Damerow S. (2022). Depressive Symptomatik in der Allgemeinbevölkerung vor und im ersten Jahr der COVID-19-Pandemie: Ergebnisse der GEDA-Studie 2019, 2020. J. Health Monitor. 4, 3–23. doi: 10.25646/10664 PMC9838134 36654684

[ref30] He C. Levis B. Riehm K. E. Saadat N. Levis A. W. Azar M.. (2020). The accuracy of the patient health Questionnaire-9 algorithm for screening to detect major depression: an individual participant data Meta-analysis. Psychother. Psychosom. 89, 25–37. doi: 10.1159/000502294 31593971 PMC6960351

[ref31] Hinz A. Mehnert A. Kocalevent R.-D. Brähler E. Forkmann T. Singer S.. (2016). Assessment of depression severity with the PHQ-9 in cancer patients and in the general population. BMC Psychiatry 16:22. doi: 10.1186/s12888-016-0728-6, PMID: 26831145 PMC4736493

[ref32] Hoebel J. Müters S. Kuntz B. Lange C. Lampert T. (2015). Messung des subjektiven sozialen Status in der Gesundheitsforschung mit einer deutschen Version der MacArthur Scale. Bundesgesundheitsblatt Gesundheitsforschung Gesundheitsschutz 58, 749–757. doi: 10.1007/s00103-015-2166-x, PMID: 25986532

[ref33] Ingram R. E. Luxton D. D. (2005). “Vulnerability-stress models” in Development of psychopathology: A vulnerability-stress perspective. eds. Hankin B. Abela J. (Cham: SAGE Publications, Inc), 32–46.

[ref34] Jacobi F. Höfler M. Siegert J. Mack S. Gerschler A. Scholl L.. (2014). Twelve-month prevalence, comorbidity and correlates of mental disorders in Germany: the mental health module of the German health interview and examination survey for adults (DEGS1-MH). Int. J. Methods Psychiatr. Res. 23, 304–319. doi: 10.1002/mpr.1439, PMID: 24729411 PMC6878234

[ref35] Johnson D. Dupuis G. Piche J. Clayborne Z. Colman I. (2018). Adult mental health outcomes of adolescent depression: a systematic review. Depress. Anxiety 35, 700–716. doi: 10.1002/da.22777 29878410

[ref36] Jordan S. Hoebel J. (2015). Gesundheitskompetenz von Erwachsenen in Deutschland: Ergebnisse der Studie "Gesundheit in Deutschland aktuell" (GEDA). Bundesgesundheitsblatt Gesundheitsforschung Gesundheitsschutz 58, 942–950. doi: 10.1007/s00103-015-2200-z, PMID: 26227894

[ref37] Kang H.-J. Kim S.-Y. Bae K.-Y. Kim S.-W. Shin I.-S. Yoon J.-S.. (2015). Comorbidity of depression with physical disorders: research and clinical implications. Chonnam Med. J. 51, 8–18. doi: 10.4068/cmj.2015.51.1.8, PMID: 25914875 PMC4406996

[ref38] Kendall K. M. van Assche E. Andlauer T. F. M. Choi K. W. Luykx J. J. Schulte E. C.. (2021). The genetic basis of major depression. Psychol. Med. 51, 2217–2230. doi: 10.1017/S0033291721000441 33682643

[ref39] Kocalevent R.-D. Hinz A. Brähler E. (2013). Standardization of the depression screener patient health questionnaire (PHQ-9) in the general population. Gen. Hosp. Psychiatry 35, 551–555. doi: 10.1016/j.genhosppsych.2013.04.006 23664569

[ref40] Köhler C. A. Evangelou E. Stubbs B. Solmi M. Veronese N. Belbasis L.. (2018). Mapping risk factors for depression across the lifespan: an umbrella review of evidence from meta-analyses and Mendelian randomization studies. J. Psychiatr. Res. 103, 189–207. doi: 10.1016/j.jpsychires.2018.05.020, PMID: 29886003

[ref41] König L. Breves P. Linnemann G. A. Hamer T. Suhr R. (2024a). Climate change distress and impairment in Germany. Front. Public Health 12:1432881. doi: 10.3389/fpubh.2024.1432881, PMID: 39381767 PMC11458408

[ref42] König H. König H.-H. Konnopka A. (2019). The excess costs of depression: a systematic review and meta-analysis. Epidemiol. Psychiatr. Sci. 29:e30. doi: 10.1017/s2045796019000180, PMID: 30947759 PMC8061284

[ref43] König L. Schröder R. Hamer T. Suhr R. (2024b). Eating disorders and health literacy in Germany: results from two representative samples of adolescents and adults. Front. Psychol. 15:1464651. doi: 10.3389/fpsyg.2024.1464651, PMID: 39351107 PMC11439665

[ref44] Kroenke K. Spitzer R. L. Williams J. B. (2001). The PHQ-9: validity of a brief depression severity measure. J. Gen. Intern. Med. 16, 606–613. doi: 10.1046/j.1525-1497.2001.016009606.x, PMID: 11556941 PMC1495268

[ref45] Kroenke K. Spitzer R. L. Williams J. B. W. Löwe B. (2010). The patient health questionnaire somatic, anxiety, and depressive symptom scales: a systematic review. Gen. Hosp. Psychiatry 32, 345–359. doi: 10.1016/j.genhosppsych.2010.03.006, PMID: 20633738

[ref46] Lee F. S. Heimer H. Giedd J. N. Lein E. S. Šestan N. Weinberger D. R.. (2014). Mental health. Adolescent mental health--opportunity and obligation. Science (New York, N.Y.) 346, 547–549. doi: 10.1126/science.1260497 25359951 PMC5069680

[ref47] Levis B. Benedetti A. Ioannidis J. P. A. Sun Y. Negeri Z. He C.. (2020). Patient health Questionnaire-9 scores do not accurately estimate depression prevalence: individual participant data meta-analysis. J. Clin. Epidemiol. 122, 115–128.e1. doi: 10.1016/j.jclinepi.2020.02.002 32105798

[ref48] Levis B. Benedetti A. Thombs B. D. (2019). Accuracy of patient health Questionnaire-9 (PHQ-9) for screening to detect major depression: individual participant data meta-analysis. BMJ 365:l1476. doi: 10.1136/bmj.l1476, PMID: 30967483 PMC6454318

[ref49] Lim G. Y. Tam W. W. Lu Y. Ho C. S. Zhang M. W. Ho R. C. (2018). Prevalence of depression in the community from 30 countries between 1994 and 2014. Sci. Rep. 8:2861. doi: 10.1038/s41598-018-21243-x, PMID: 29434331 PMC5809481

[ref50] Lincoln A. Paasche-Orlow M. K. Cheng D. M. Lloyd-Travaglini C. Caruso C. Saitz R.. (2006). Impact of health literacy on depressive symptoms and mental health-related: quality of life among adults with addiction. J. Gen. Intern. Med. 21, 818–822. doi: 10.1111/j.1525-1497.2006.00533.x, PMID: 16881940 PMC1831585

[ref51] Liu Q. He H. Yang J. Feng X. Zhao F. Lyu J. (2020). Changes in the global burden of depression from 1990 to 2017: findings from the global burden of disease study. J. Psychiatr. Res. 126, 134–140. doi: 10.1016/j.jpsychires.2019.08.002 31439359

[ref52] Mallia L. Chirico A. Zelli A. Galli F. Palombi T. Bortoli L.. (2020). The implementation and evaluation of a media literacy intervention about PAES use in sport science students. Front. Psychol. 11:368. doi: 10.3389/fpsyg.2020.00368, PMID: 32265771 PMC7105711

[ref53] Martin A. Rief W. Klaiberg A. Braehler E. (2006). Validity of the brief patient health questionnaire mood scale (PHQ-9) in the general population. Gen. Hosp. Psychiatry 28, 71–77. doi: 10.1016/j.genhosppsych.2005.07.003 16377369

[ref54] Maske U. E. Busch M. A. Jacobi F. Beesdo-Baum K. Seiffert I. Wittchen H.-U.. (2015). Current major depressive syndrome measured with the patient health Questionnaire-9 (PHQ-9) and the composite international diagnostic interview (CIDI): results from a cross-sectional population-based study of adults in Germany. BMC Psychiatry 15:77. doi: 10.1186/s12888-015-0463-4, PMID: 25884294 PMC4394554

[ref55] Mo P. K. H. Xie L. Mak W. W. S. (2023). Is inadequate health literacy associated with worse health outcomes among Chinese individuals with depression? Health Promot. Int. 38:3. doi: 10.1093/heapro/daad042, PMID: 37279468

[ref56] Moor I. Herke M. Markert J. Böhm M. Reiß F. Bilz L.. (2024). Trends in health inequalities in childhood and adolescence in Germany: results of the HBSC study 2009, 10 - 2022. J. Health Monitor. 9, 79–98. doi: 10.25646/11876, PMID: 38559681 PMC10977468

[ref57] Moreno-Agostino D. Wu Y.-T. Daskalopoulou C. Hasan M. T. Huisman M. Prina M. (2021). Global trends in the prevalence and incidence of depression:a systematic review and meta-analysis. J. Affect. Disord. 281, 235–243. doi: 10.1016/j.jad.2020.12.035, PMID: 33338841

[ref58] Nutbeam D. (1998). Health promotion glossary. Health Promot. Int. 13, 349–364. doi: 10.1093/heapro/13.4.349

[ref59] Otte C. Gold S. M. Penninx B. W. Pariante C. M. Etkin A. Fava M.. (2016). Major depressive disorder. Nat. Rev. Dis. Primers 2:16065. doi: 10.1038/nrdp.2016.65 27629598

[ref60] Paasche-Orlow M. K. Wolf M. S. (2007). The causal pathways linking health literacy to health outcomes. Am. J. Health Behav. 31, 19–26. doi: 10.5993/AJHB.31.s1.4 17931132

[ref61] Pelikan J. M. Ganahl K. van den Broucke S. Sørensen K. (2019). “Measuring health literacy in Europe: introducing the European health literacy survey questionnaire (HLS-EU-Q)” in International handbook of health literacy. Research, practice and policy across the life-span. eds. Bauer U. Levin-Zamir D. Okan O. (Bristol: Policy Press), 115–138.

[ref62] Pelikan J. M. Röthlin F. Ganahl K. (2014). Measuring comprehensive health literacy in general populations: Validation of instrument, indices and scales of the HLS-EU study. Maryland, USA: Bethesda.

[ref63] Plass D. Vos T. Hornberg C. Scheidt-Nave C. Zeeb H. Krämer A. (2014). Trends in disease burden in Germany: results, implications and limitations of the global burden of disease study. Deutsches Arzteblatt Int. 111, 629–638. doi: 10.3238/arztebl.2014.0629, PMID: 25316518 PMC4199248

[ref64] Porst M. Lippe E. Leddin J. Anton A. Wengler A. Breitkreuz J.. (2022). The burden of disease in Germany at the national and regional level. Deutsches Arzteblatt Int. 119, 785–792. doi: 10.3238/arztebl.m2022.0314, PMID: 36350160 PMC9902892

[ref65] Richardson L. P. McCauley E. Grossman D. C. McCarty C. A. Richards J. Russo J. E.. (2010). Evaluation of the patient health Questionnaire-9 item for detecting major depression among adolescents. Pediatrics 126, 1117–1123. doi: 10.1542/peds.2010-0852, PMID: 21041282 PMC3217785

[ref66] Shevlin M. Butter S. McBride O. Murphy J. Gibson-Miller J. Hartman T. K.. (2022). Measurement invariance of the patient health questionnaire (PHQ-9) and generalized anxiety disorder scale (GAD-7) across four European countries during the COVID-19 pandemic. BMC Psychiatry 22:154. doi: 10.1186/s12888-022-03787-5, PMID: 35232409 PMC8886334

[ref67] Shorey S. Ng E. D. Wong C. H. J. (2022). Global prevalence of depression and elevated depressive symptoms among adolescents: a systematic review and meta-analysis. Br. J. Clin. Psychol. 61, 287–305. doi: 10.1111/bjc.12333, PMID: 34569066

[ref68] Smith S. A. Moore E. J. (2012). Health literacy and depression in the context of home visitation. Matern. Child Health J. 16, 1500–1508. doi: 10.1007/s10995-011-0920-8, PMID: 22120425 PMC3443343

[ref69] Sørensen K. van den Broucke S. Pelikan J. M. Fullam J. Doyle G. Slonska Z.. (2013). Measuring health literacy in populations: illuminating the design and development process of the European health literacy survey questionnaire (HLS-EU-Q). BMC Public Health 13:948. doi: 10.1186/1471-2458-13-948, PMID: 24112855 PMC4016258

[ref70] Spitzer R. L. Kroenke K. Williams J. B. (1999). Validation and utility of a self-report version of PRIME-MD: the PHQ primary care study. Primary care evaluation of mental disorders. Patient health questionnaire. JAMA 282, 1737–1744. doi: 10.1001/jama.282.18.1737 10568646

[ref71] Stocker R. Tran T. Hammarberg K. Nguyen H. Rowe H. Fisher J. (2021). Patient health questionnaire 9 (PHQ-9) and general anxiety disorder 7 (GAD-7) data contributed by 13,829 respondents to a national survey about COVID-19 restrictions in Australia. Psychiatry Res. 298:113792. doi: 10.1016/j.psychres.2021.113792, PMID: 33592399 PMC9754708

[ref72] Sullivan P. F. Neale M. C. Kendler K. S. (2000). Genetic epidemiology of major depression: review and meta-analysis. Am. J. Psychiatry 157, 1552–1562. doi: 10.1176/appi.ajp.157.10.1552, PMID: 11007705

[ref73] Tassone V. K. Duffy S. F. Dunnett S. Boparai J. K. Valentina Z. C. Jung H.. (2024). Decreased odds of depressive symptoms and suicidal ideation with higher education, depending on sex and employment status. PLoS One 19:e0299817. doi: 10.1371/journal.pone.0299817 38568884 PMC10990184

[ref74] Torsheim T. Cavallo F. Levin K. A. Schnohr C. Mazur J. Niclasen B.. (2016). Psychometric validation of the revised family affluence scale: a latent variable approach. Child Indic. Res. 9, 771–784. doi: 10.1007/s12187-015-9339-x, PMID: 27489572 PMC4958120

[ref75] Tüzün H. Demirköse H. Özkan S. İlhan M. N. (2021). Socioeconomic factors related to prevalence, severity, and contact coverage of depression in primary health care. Psychiatry Clin. Psychopharmacol. 31, 457–467. doi: 10.5152/pcp.2021.21051 38765649 PMC11079699

[ref76] Uemura K. Yamada M. Okamoto H. (2021). The effectiveness of an active learning program in promoting a healthy lifestyle among older adults with low health literacy: a randomized controlled trial. Gerontology 67, 25–35. doi: 10.1159/000511357 33271536

[ref77] University Medicine Greifswald (2024). Family affluence scale. Available at: https://www.euthyroid.eu/frageboegen/ (Accessed July 12, 2024).

[ref78] van Zoonen K. Buntrock C. Ebert D. D. Smit F. Reynolds C. F. Beekman A. T. F.. (2014). Preventing the onset of major depressive disorder: a meta-analytic review of psychological interventions. Int. J. Epidemiol. 43, 318–329. doi: 10.1093/ije/dyt175, PMID: 24760873 PMC4023317

[ref79] Vos T. Allen C. Arora M. Barber R. M. Bhutta Z. A. Brown A.. (2016). Global, regional, and national incidence, prevalence, and years lived with disability for 310 diseases and injuries, 1990-2015: a systematic analysis for the global burden of disease study 2015. Lancet 388, 1545–1602. doi: 10.1016/S0140-6736(16)31678-6, PMID: 27733282 PMC5055577

[ref80] Walters R. Leslie S. J. Polson R. Cusack T. Gorely T. (2020). Establishing the efficacy of interventions to improve health literacy and health behaviours: a systematic review. BMC Public Health 20:1040. doi: 10.1186/s12889-020-08991-0, PMID: 32605608 PMC7329558

[ref81] Wang X. Cheng Z. (2020). Cross-sectional studies: strengths, weaknesses, and recommendations. Chest 158, S65–S71. doi: 10.1016/j.chest.2020.03.012 32658654

[ref82] Wetherall K. Robb K. A. O'Connor R. C. (2019). Social rank theory of depression: a systematic review of self-perceptions of social rank and their relationship with depressive symptoms and suicide risk. J. Affect. Disord. 246, 300–319. doi: 10.1016/j.jad.2018.12.045, PMID: 30594043

[ref83] Whooley M. A. Wong J. M. (2013). Depression and cardiovascular disorders. Annu. Rev. Clin. Psychol. 9, 327–354. doi: 10.1146/annurev-clinpsy-050212-185526 23537487

[ref84] World Health Organization (2008). The global burden of disease. 2004 update. Geneva: World Health Organization.

[ref85] World Health Organization (2019). International classification of diseases, eleventh revision (ICD-11): The global standard for diagnostic health information. Geneva: World Health Organization.

[ref86] Zhang X. C. Kuchinke L. Woud M. L. Velten J. Margraf J. (2017). Survey method matters: online, offline questionnaires and face-to-face or telephone interviews differ. Comput. Hum. Behav. 71, 172–180. doi: 10.1016/j.chb.2017.02.006

