# Supplementary material for: Depression and health literacy among adolescents and adults in Germany: findings from two representative samples
Source: Front Psychol. 2024 Dec 11;15:1494333. doi: 10.3389/fpsyg.2024.1494333 (PMC11669750; doi:10.3389/fpsyg.2024.1494333)
Supplement: Supplementary file 1 [file Data_Sheet_1.pdf]

Supplement

Depression and health literacy among adolescents and adults in Germany: findings from two representative samples

Lars König[1,2†]*, Rebekka Schröder[1†], Tim Hamer[1], Ralf Suhr[1,2]

[1]Stiftung Gesundheitswissen, Berlin, Germany

[2]Institut für Medizinische Soziologie und Rehabilitationswissenschaft, Charité – Universitätsmedizin Berlin, Berlin, Germany

† These authors contributed equally to this work and share first authorship.

*** Correspondence:**
Dr. rer. nat. Lars König
lars.koenig@stiftung-gesundheitswissen.de

Supplementary Table 1: Individual item responses for the PHQ-9 questionnaire in the weighted adult sample.

Items	Not at all	Several days	More than half the days	Nearly every day	Missing value
Little interest or pleasure in doing things	48.4%	40.9%	7.5%	3.1%	0.1%
Feeling down, depressed, or hopeless	54.4%	35.3%	6.7%	3.6%	0.1%
Trouble falling or staying asleep, or sleeping too much	29.7%	44.7%	13.8%	11.7%	0.1%
Feeling tired or having little energy	21.2%	55.1%	14.1%	9.5%	0.1%
Poor appetite or overeating	62.7%	28.6%	6.0%	2.7%	0.1%
Feeling bad about yourself — or that you are a failure or have let yourself or your family down	66.9%	24.1%	5.8%	3.1%	0.1%
Trouble concentrating on things, such as reading the newspaper or watching television	52.1%	37.9%	6.9%	3.1%	0.1%
Moving or speaking so slowly that other people could have noticed? Or the opposite — being so fidgety or restless that you have been moving around a lot more than usual	82.6%	13.2%	3.2%	0.8%	0.2%
Thoughts that you would be better off dead or of hurting yourself in some way	86.6%	10.5%	1.4%	1.3%	0.1%

Supplementary Table 2: Individual item responses for the PHQ-9 questionnaire in the weighted adolescent sample.

Items	Not at all	Several days	More than half the days	Nearly every day	Missing value
Little interest or pleasure in doing things	50.5%	36.9%	8.8%	3.8%	0.0%
Feeling down, depressed, or hopeless	63.0%	26.5%	6.8%	3.7%	0.0%
Trouble falling or staying asleep, or sleeping too much	57.6%	28.2%	9.0%	5.2%	0.0%
Feeling tired or having little energy	44.0%	37.9%	10.9%	7.2%	0.0%
Poor appetite or overeating	64.0%	23.6%	7.8%	4.6%	0.0%
Feeling bad about yourself — or that you are a failure or have let yourself or your family down	60.3%	26.6%	9.2%	3.9%	0.0%
Trouble concentrating on things, such as reading the newspaper or watching television	49.4%	37.0%	9.8%	3.9%	0.0%
Moving or speaking so slowly that other people could have noticed? Or the opposite — being so fidgety or restless that you have been moving around a lot more than usual	75.5%	15.9%	6.5%	2.0%	0.0%
Thoughts that you would be better off dead or of hurting yourself in some way	80.5%	12.3%	5.4%	1.8%	0.0%
